# Retraction: Genetic Variation in the Androgen Receptor and Measures of Plasma Testosterone Levels Suggest Androgen Dysfunction in Alzheimer's Disease

**DOI:** 10.3389/fnins.2019.01265

**Published:** 2019-11-20

**Authors:** Frontiers Editorial Office

The Journal and Chief Editors retract the August 2018 article cited above.

After publication, the authors were made aware of an unfortunate coding error resulting in the data analyzed in this study being inconsistent with the data provided in the Alzheimer's Disease Neuroimaging Initiative repository. Due to this honest error, the authors request that this article be retracted.

The retraction of the article was approved by the Chief Editors of Frontiers in Neuroscience, Neurodegeneration and the Editor-in-Chief of Frontiers.

